# Variability in stem taper surface topography affects the degree of corrosion and fretting in total hip arthroplasty

**DOI:** 10.1038/s41598-021-88234-3

**Published:** 2021-04-30

**Authors:** Kilian Elia Stockhausen, Christoph Riedel, Alex Victoria Belinski, Dorothea Rothe, Thorsten Gehrke, Felix Klebig, Matthias Gebauer, Michael Amling, Mustafa Citak, Björn Busse

**Affiliations:** 1 Department of Osteology and Biomechanics, University Medical Center Hamburg-Eppendorf, Lottestr. 55a, 22529 Hamburg, Germany; 2 Department of Mechanical Engineering, University of California, 6141 Etcheverry Hall, Berkeley, CA USA; 3 Department of Orthopedic Surgery, Helios ENDO-Klinik, Holstenstraße 2, 22767 Hamburg, Germany; 4 Interdisciplinary Competence Center for Interface Research (ICCIR), Martinistr. 52, 20251 Hamburg, Germany; 5 Forum Medical Technology Health Hamburg (FMTHH), Butenfeld 34, 22529 Hamburg, Germany

**Keywords:** Implants, Quality of life, Orthopaedics

## Abstract

Degradation at the modular head-neck interface in total hip arthroplasty (THA) is predominately expressed in the form of corrosion and fretting, potentially causing peri-prosthetic failure by adverse reactions to metal debris. This retrieval study aimed to quantify variations in stem taper surface topographies and to assess the influence on the formation of corrosion and/or fretting in titanium alloy stem tapers combined with metal and ceramic heads. Four hip stem designs (Alloclassic, CLS, Bicontact and SL-Plus) were characterized using high-resolution 3D microscopy, and corrosion and fretting were rated using the Goldberg scoring scheme. Quantification of the taper surface topographies revealed a high variability in surface characteristics between threaded stem tapers: Alloclassic and CLS tapers feature deeply threaded trapezoid-shaped profiles with thread heights over 65 µm. The sawtooth-shaped Bicontact and triangular SL-Plus taper are characterized by low thread heights below 14 µm. Significantly lower corrosion and fretting scores were observed in lightly threaded compared to deeply threaded tapers in ceramic head combinations. No significant differences in corrosion or fretting scores with thread height were found in pairings with metal heads. Understanding the relationship between stem taper surface topography and the formation of corrosion and fretting could help to improve the performance of modern THAs and lead to longer-lasting clinical results.

## Introduction

The advancements in modern total hip arthroplasty (THA) towards modularity are highly valued by surgeons. The surgeons are provided with the tools to better fit the anatomy of specific patients while maintaining intraoperative flexibility. Modular hip implants allow the surgeon to match the specific hip biomechanics and adjust for patient-specific factors like leg length or offset. Despite the apparent benefits of modular THA, the need for revision procedures remains a considerable problem and the number of hip revision procedures is projected to double by the year 2026^1^. As revision THA patients are associated with longer hospital stays, higher complications rates, and higher in-hospital mortality rates, understanding the causes of THA failure is paramount to improve the functional outcome and longevity of the prostheses^2^. The reasons for THA failure are multi-factorial and include both patient- and implant-related factors as well as failures related to inadequate surgical technique^3,4^. In recent years degradation of the femoral head-neck interface by fretting and corrosion damage^5,6^ has emerged as another impact factor contributing to THA failure and is estimated to account for up to 3% of all THA revision procedures^7^. It is associated with the release of metal ions into the peri-prosthetic tissue, potentially leading to implant failure through adverse reactions to metal debris^8–11^. The exact causes of the degradation process remain unclear but several influencing factors have been suggested to contribute including the body mass index^12^, time in situ^13^, mixing of alloys^14,15^, the femoral head size^16,17^, the flexural rigidity^18^, the female taper angle^17^ and taper angle mismatch^19^, the taper length^12^ and diameter^20^ as well as the stem surface roughness^21–27^.

Ceramic femoral heads are extensively used due to low wear characteristics^28^ and in response to controversial complications in metal-on-metal bearings^29,30^. The use of ceramic heads has also guided the design of stem tapers: due to the brittle nature of ceramics, the head and stem taper angle are chosen such that most contact occurs at the center of the head to minimize stresses and prevent burst fracture^31–33^. Additionally, a threaded surface topography was introduced to distribute loading forces during assembly more efficiently and reduce local contact stresses^33,34^. Even though the threaded design was initially introduced with the rise of ceramic heads they are now also commonly applied in combination with metal heads^33^.

There is a large variability in stem taper designs that differ in terms of their taper length, distal and proximal base diameter, taper angle as well as roundness and straightness. This even applies to stem taper designs that share the same size designation, e.g., “12/14”. Not all hip stems labelled as “12/14” are uniform but show considerable variations both in their taper geometry as well as surface topography which can be smooth or threaded to a varying degree^35,36^. Previous studies investigated the effect of smooth or micro-threaded taper surfaces on the degree of corrosion and fretting damage. Kop et al*.* reported that CoCr alloy necks with a micro-threaded finish were more corroded compared to smooth CoCr stems but found the opposite trend with Ti-6Al-4 V alloy necks^37^. Brock et al*.* as well as Hothi et al*.* compared shorter, threaded 12/14 tapers with longer, smooth 11/13 tapers and reported higher wear rates and material loss with threaded surface finishes^38, 39^. Arnholt et. al. compared smooth and micro-threaded stem tapers fabricated of CoCr and Ti-6Al-4 V matched with CoCr femoral heads and found no link to increased fretting corrosion damage or material release^22^. Kurtz et al*.* found less fretting and corrosion with ceramic compared to CoCr heads, but noted that the lower scores cannot be attributed to differences in surface topography^28^. Goldberg et al*.* stated that longer times in situ are associated with increased neck corrosion, while neck fretting appears unaffected by it^13^. Contrarily, other studies concluded that there is no correlation between the time in situ and corrosion and fretting damage. They found other factors to have a stronger effect on damage accumulation such as an increased femoral head size^16^ or a larger taper diameter with increased head-neck contact surface^20^. However, Triantafyllopoulos et al*.* reported that the head size does not influence either fretting or corrosion at the head-neck modular junction^40^. Clearly, the available published literature regarding the contribution of stem taper surface topography on corrosion and fretting damage is partially contradictory and remains complex.

So far, most studies focused on a comparison between smooth and micro-threaded surface finishes (thread height < 20 µm)^21–25^. In the present study we aimed to further quantify the variability in taper surface topographies of hip stems that are commercially available (Alloclassic, CLS, Bicontact and SL-Plus) and investigate the influence of varying surface finishes on the formation of fretting and corrosion in the head-neck interface. Additionally, the degree of corrosion and fretting is compared between ceramic and metal femoral heads with simultaneous consideration of the surface finish.

## Methods

In this retrospective retrieval study, 46 stem tapers (12/14 design, uncemented fixation) with threaded surface finishes in combination with metal and ceramic heads were analyzed. Four different stem designs were included in the study: Alloclassic (Zimmer, Winterthur, Switzerland), CLS (Zimmer, Warsaw, Indiana, USA), Bicontact (B.Braun-Aesculap, Tuttlingen, Germany) and SL-Plus (Smith & Nephew, Baar, Switzerland). The implants were collected in revision surgeries at the HELIOS ENDO-Klinik Hamburg for septic (n = 25) and aseptic (n = 21) loosening, washed with water and subsequently placed in an autoclave for sterilization. All stems are fabricated from a titanium alloy (Alloclassic/CLS/SL-Plus: Ti-6Al-7Nb, Bicontact Ti-6AI-4 V). After approval by the ethics committee of the General Medical Council Hamburg, the written informed consent by the patients to be included in the study was obtained. Subsequently, pseudonymized clinical data were gathered. The stems were retrieved from 24 men and 22 women with similar age, BMI and time in situ. Patient demographics and implant characteristics are listed in Table 1. All experiments were performed in accordance with the relevant guidelines and regulations.

**Table 1:** Implant characteristics and patient demographics included in the retrieval analysis.

Hip stem	Frequency (#)	Sex (male/female)	Age (years)	BMI (#)	Time in situ (years)	Femoral head (metal/ceramics)	Revision reason (septic/aseptic)
Alloclassic	7	3/4	60.1 ± 8.1	27.1 ± 5.8	5.71 ± 0.95	3/4	3/4
CLS	13	10/3	62.2 ± 11.2	28.8 ± 4.9	7.08 ± 4.54	6/7	10/3
Bicontact	12	4/8	64.7 ± 6.2	28.3 ± 3.8	6.58 ± 3.68	9/3	7/5
SL-Plus	14	7/7	64.2 ± 8.6	28.5 ± 4.2	9.00 ± 3.70	4/10	5/9
Total	46	24/22	63.2 ± 8.7	28.3 ± 4.5	7.33 ± 3.78	22/24	25/21

**Figure 1:**
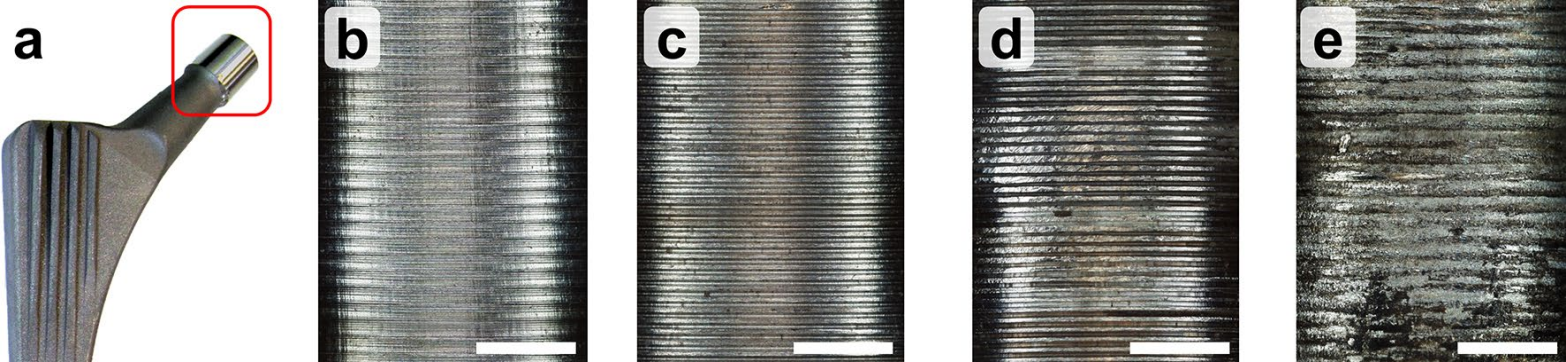
(**a**) Corrosion and fretting scores were assessed at the stem taper. Representative micrographs showing (**b**) no, (**c**) mild, (**d**) moderate and (**e**) severe damage in terms of corrosion and fretting. Scale bar is 1 mm.

Corrosion and fretting damage were assessed using an established scoring scheme published by Goldberg et al*.*^13^. Based on visual inspection under an optical stereomicroscope the tapers were rated on a 4-point scale, with a score of 1 indicating no fretting or corrosion (No visible corrosion observed; no visible signs of fretting observed); 2, indicating mild damage (< 30% of taper surface discolored or dull; single band or bands of fretting scars involving three or fewer machine lines on taper surface); 3, indicating moderate damage (> 30% of taper surface discolored or dull, < 10% of taper surface containing black debris, pits, or etch marks; several bands of fretting scars or single band involving more than three machine lines); and 4, indicating severe damage (> 10% of taper surface containing black debris, pits, or etch marks; several bands of fretting scars involving several adjacent machine lines, or flattened areas with nearby fretting scars) (Fig. 1). Using the adaptation of Triantafyllopoulus et al., each taper was scored in eight regions: in the distal and proximal end of the head-neck contact region along the anterior, inferior, superior and posterior axis. Fretting and corrosion scores were added resulting in a maximum score of 32^40^. The scoring scheme has been applied in several studies^16,20,22,41^ and provides both repeatable and reproducible results^42^. Each taper was scored independently by two investigators to ensure consistency of results. The scores were averaged to provide a mean score for both fretting and corrosion severity.

**Figure 2:**
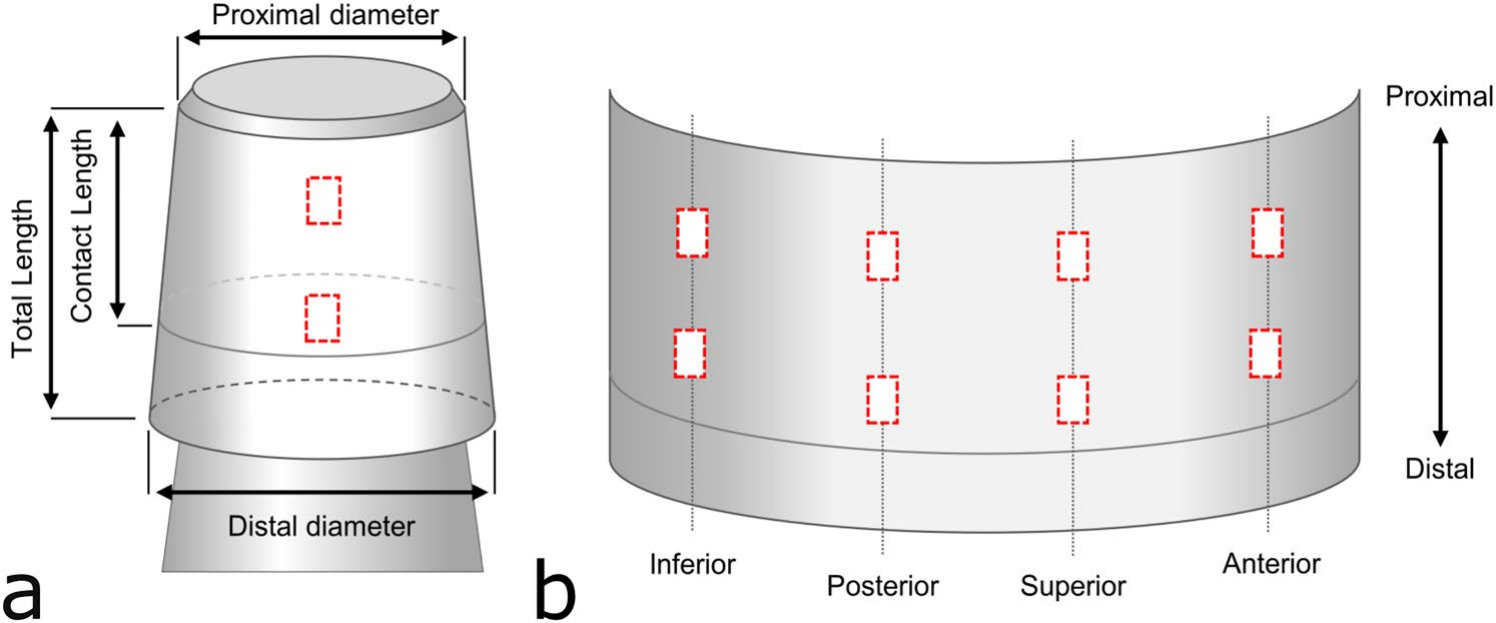
(**a**) Taper dimensions were assessed under 5 × magnification and (**b**) eight regions of interest (red boxes) in the head-neck contact region were evaluated to assess the micro-scale surface topography.

High-resolution opto-digital 3D microscopy (Olympus DSX500, Hamburg, Germany) was used to assess the surface topography. One taper of each design with a low Goldberg score was exemplarily measured at 32 points to assess the intra-taper profile variability. Four measurements along the proximal–distal axis were taken every 45-degree rotation using a 50 × objective lens with a lateral resolution of 0.5 µm and z-resolution of 0.48 µm. Based on the results it was decided that for subsequent data collection each taper would be assessed in the eight regions where Goldberg scores were collected to obtain representative values (Fig. 2). In case the head and stem taper were not in full contact, corrosion and fretting scores were collected in the contact region. The surface topography was characterized regarding the thread height, thread width and effective surface area enlargement. The thread height was defined as the difference of the maximum value of the peak height and the minimum value of valley depth. Five peaks and five valleys were selected to calculate the mean thread height. Taper dimensions (taper length, contact length, proximal and distal diameter) were measured using a 5 × objective lens and used to calculate the flexural rigidity using the equation^13^

$$D=EI=E\frac{\pi *{d}^{4}}{64}$$

where E is the Young’s modulus of the titanium alloy (110GPa for Ti-6Al-7Nb and 112GPa for Ti-6AI-4V)^18,43^, I the area moment of inertia and d the diameter of the stem at the contact point with the head taper.

Shapiro–Wilk tests were used to check for normality. An analysis of covariance (ANCOVA) with appropriate post-hoc tests (Bonferroni or Games Howell) was performed to detect differences in corrosion and fretting scores between implant designs and taper surface topographies, while checking for potential covariates (BMI, contact length of the taper, time in situ, flexural rigidity and head diameter). Spearman’s rank correlations were used to check for (i.) correlations between the individual covariates and (ii.) correlations between the covariates and the corrosion and fretting scores. Inter-rater agreement on corrosion and fretting scores were determined using Cohen’s kappa tests. Statistical tests were performed using SPSS with a significance level of α = 0.05.

## Results

**Figure 3:**
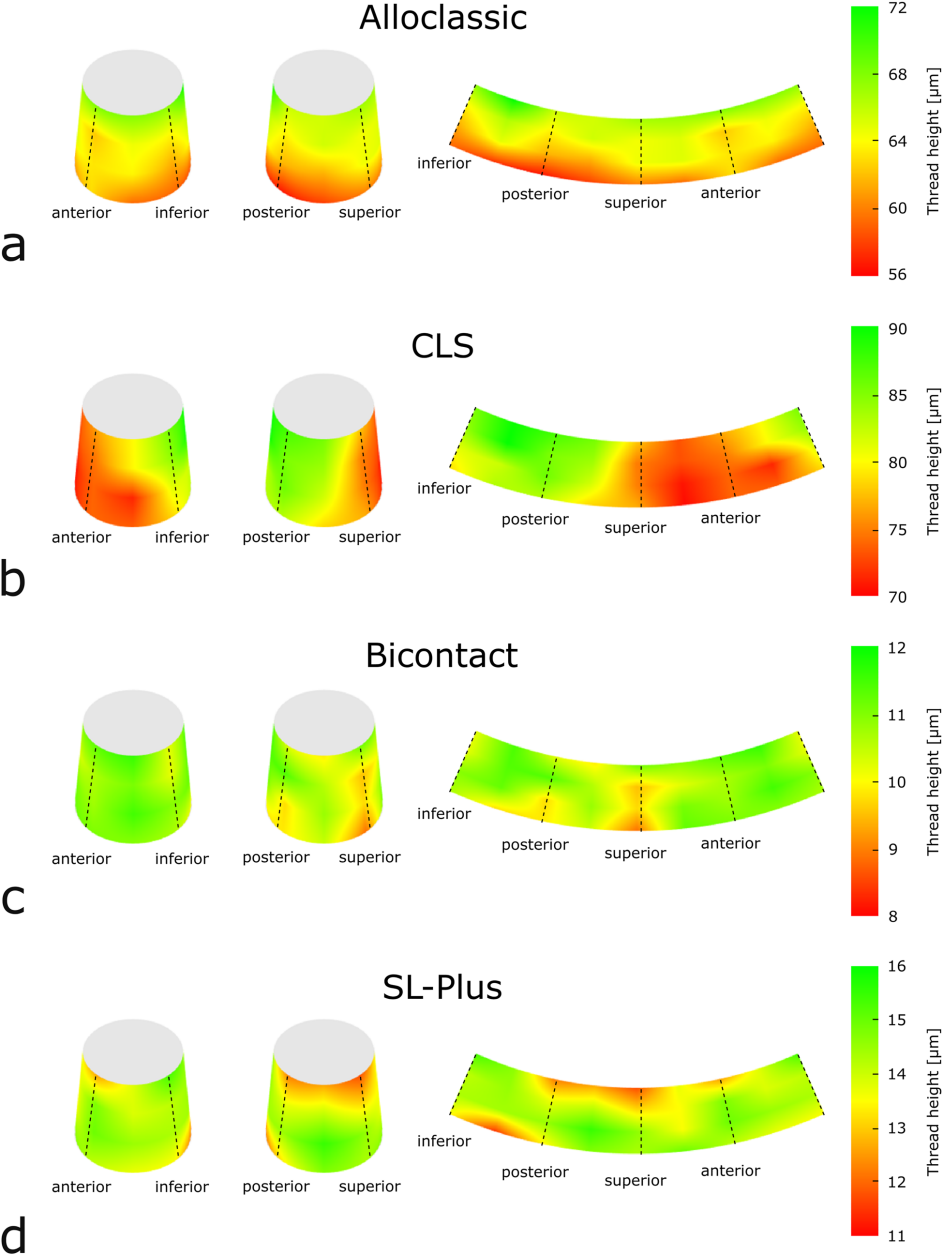
Interpolation of thread heights reveals intra-stem inhomogeneity throughout all stem tapers.

**Figure 4:**
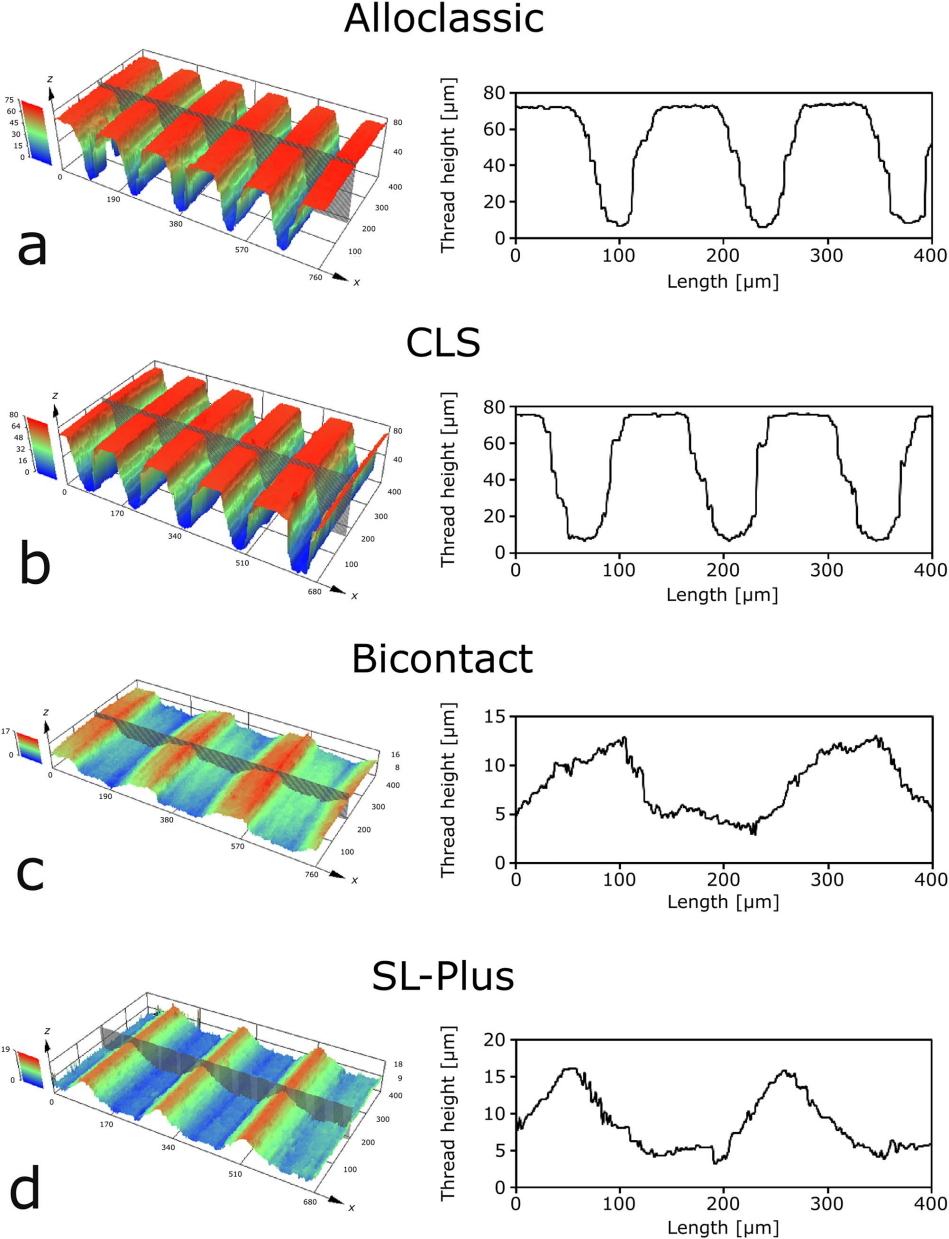
Two distinct groups of surface topography were identified: the Alloclassic and CLS taper are characterized by deep, trapezoidal threads while the sawtooth-shaped Bicontact and triangular SL-Plus display low thread heights.

High resolution 3D microscopy reveals considerable intra-implant and inter-group differences in stem taper surface topography regarding the thread height, thread width and profile shape. Across the surface of individual stem tapers substantial differences in thread height were measured for all designs (Fig. 3). Exemplary thread height measurements of selected stem tapers reveal deviations of up to 27.1% (CLS, range: 67.6–92.7 µm) between the highest and the lowest point. The Alloclassic taper is characterized by 21.5% (55.8–71.1 µm), the Bicontact by 23.3% (8.9–11.6 µm) and the SL-Plus by 25.2% (11.6–15.5 µm) thread height variations. Beyond the distinct differences in thread heights, the profile shape also differed substantially: both deeply threaded taper designs (Alloclassic and CLS) portray trapezoidal profiles with mean thread heights of 65.6 ± 3.6 µm and 68.9 ± 13.9 µm, respectively. The Bicontact stem taper is characterized by a sawtooth-like profile with a mean thread height of 10.4 ± 1.3 µm while the SL-Plus taper features a triangular profile (13.4 ± 2.3 µm) (Fig. 4). The thread width is almost identical for the deeply threaded tapers with 140.2 ± 0.3 µm (Alloclassic) and 140.1 ± 0.5 µm (CLS). Comparing the thread widths of the lightly threaded Bicontact and SL-Plus stem tapers shows significant differences: with 224.8 ± 16.4 µm the Bicontact displays a significantly larger thread width compared to the SL-Plus (206.6 ± 12.2 µm). In contrast to the deeply threaded taper designs, the lightly threaded stem tapers present a larger inhomogeneity in thread width. Regardless of the thread height and profile shape all stem tapers show a consistent thread pattern, with no periodic difference in the repetition and heights of the threads.

The effective surface enlargement is dependent on the thread height and overall profile shape, and is significantly larger in the deeply threaded stem tapers (*p* < 0.001). Due to their similar profile shape, the Alloclassic and CLS stem have a comparable surface enlargement of 1.86 ± 0.06 and 1.95 ± 0.26, respectively. Despite their differing profile shape, the Bicontact and SL-Plus stem show no significant difference in surface enlargement (1.08 ± 0.03 vs. 1.10 ± 0.02). The material combination at the head-neck interface had no impact on the topographic parameters for the Alloclassic and CLS stem tapers. However, the lightly threaded stem tapers both had significantly lower thread heights when coupled with metal heads compared to ceramic heads. No significant differences were measured for the flexural rigidity between the hip stems. Detailed results of thread heights, thread widths, surface enlargements and flexural rigidities are summarized in Table 2.

**Table 2:** Stem taper characteristics sub-divided into material pairings.

Hip stem	Metal + ceramic heads	Metal heads	Ceramic heads
**Thread height **(**µm**)			
Alloclassic	65.6 (3.6)^C,D^	66.6 (3.7)^C,D^	64.8 (3.9)^C,D^
CLS	68.9 (13.9)^C,D^	70.0 (17.2)^C,D^	67.9 (11.7)^C,D^
Bicontact	10.4 (1.3)^A,B,D^	9.9 (1.0)^A,B^	11.9 (0.5)^A,B,^*
SL-Plus	13.4 (2.3)^A,B,C^	11.5 (2.4)^A,B^	14.2 (1.8)^A,B,^*
**Thread width **(**µm**)			
Alloclassic	140.2 (0.3)^C,D^	140.0 (0.4)^C,D^	140.3 (0.1)^C,D^
CLS	140.1 (0.5)^C,D^	139.9 (0.6)^C,D^	140.1 (0.3)^C,D^
Bicontact	224.8 (16.4)^A,B,D^	222.1 (17.4)^A,B^	232.7 (11.4)^A,B,D^
SL-Plus	206.6 (12.2)^A,B,C^	199.5 (19.5)^A,B^	209.5 (7.6)^A,B,C^
**Surface enlargement**			
Alloclassic	1.86 (0.06)^C,D^	1.84 (0.11)^C,D^	1.87 (0.02)^C,D^
CLS	1.95 (0.26)^C,D^	1.94 (0.34)^C,D^	1.97 (0.20)^C,D^
Bicontact	1.08 (0.03)^A,B^	1.08 (0.03)^A,B^	1.08 (0.01)^A,B^
SL-Plus	1.10 (0.02)^A,B^	1.10 (0.3)^A,B^	1.10 (0.02)^A,B^
**Flexural rigidity **(**Nm**^**2**^)			
Alloclassic	183.9 (10.2)	177.3 (8.4)	190.4 (8.0)
CLS	189.6 (11.9)	184.2 (6.8)	194.1 (13.8)
Bicontact	205.6 (22.1)	205.3 (24.9)	206.5 (14.1)
SL-Plus	183.9 (15.0)	172.8 (19.2)	188.9 (10.3)

The values are given as the mean and the standard deviation. Superscript letters denote significant differences with (A) Alloclassic, (B) CLS, (C) Bicontact and (D) SL-Plus stems. Asterisks denote significant differences in the same stem design between material pairings.

**Figure 5:**
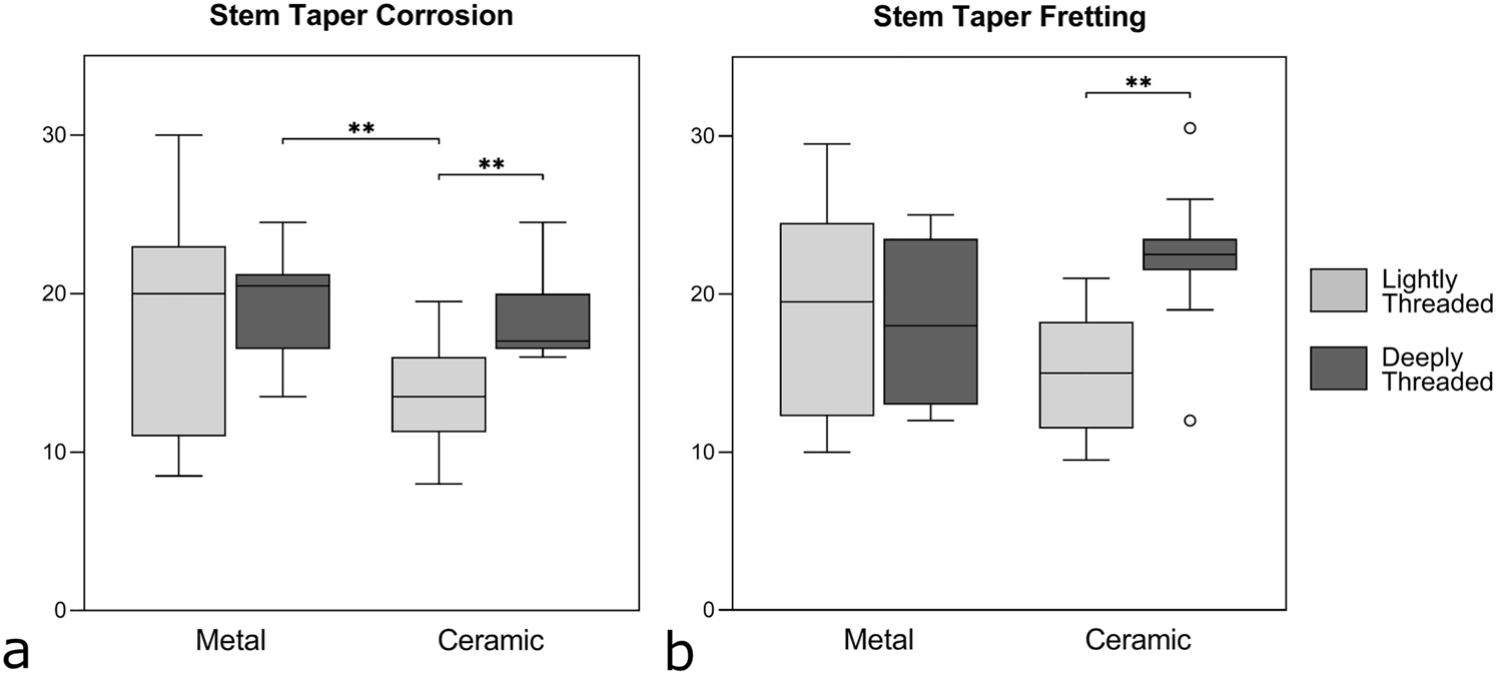
Subgroup analysis grouping lightly and deeply threaded tapers revealed (**a**) significantly lower corrosion scores for lightly threaded stem tapers in ceramic head combinations compared to deeply threaded tapers coupled with ceramic heads and deeply threaded tapers coupled with metal heads. (**b**) Lower fretting scores were assessed in lightly threaded tapers compared to deeply threaded tapers coupled with ceramic heads. No significant differences in corrosion or fretting scores with thread height were found in pairings with metal heads. The boxes display the median, the interquartile range, and the minimum as well as maximum value. Data points more distant to the median are marked as outliers. Double asterisks denote highly significant differences (*p* ≤ 0.01).

Corrosion and fretting scores for the different subgroups of deeply and lightly threaded tapers with simultaneous consideration of the head-neck material combination revealed significant differences for both corrosion (*F*^3,41^ = 4.86; *p* = 0.016) and fretting (*F*^3,41^ = 3.96; *p* = 0.014) (Fig. 5). The investigated covariates (BMI, contact length of the taper, time in situ, flexural rigidity and head diameter) showed no significant influence on the models. Pairwise comparisons revealed significantly lower corrosion for lightly threaded tapers coupled with ceramic heads (13.6 ± 3.3) compared to deeply threaded tapers coupled with ceramic heads (18.2 ± 2.6; *p* = 0.005) and compared to deeply threaded tapers coupled with metal heads (19.2 ± 3.4; *p* = 0.006). Significantly lower fretting scores were observed in lightly threaded (15.0 ± 3.8) compared to deeply threaded stem tapers in combinations with ceramic heads (22.3 ± 4.5; *p* = 0.002). No statistical differences in corrosion or fretting scores between lightly and deeply threaded tapers were found with metal heads. Corrosion scores showed no significant correlation with time in situ both for metal head (*r* = -0.19, *p* = 0.39) and ceramic head combinations (*r* = -0.03, *p* = 0.88). Also, no significant correlation between fretting scores and time in situ was found for metal heads (*r* = 0.33, *p* = 0.14) and ceramic heads (*r* = -0.11, *p* = 0.61). No significant correlation between flexural rigidity and corrosion (*r* = -0.18, *p* = 0.25) and fretting (*r* = -0.13, *p* = 0.39) was found. No association between head size and the Goldberg scores was detected for both profile types. Cohen’s kappa tests demonstrated substantial agreement between both investigators on corrosion scores (κ = 0.61) and fair agreement on fretting scores (κ = 0.39)^44^.

## Discussion

The high modularity in total hip arthroplasty enables surgeons to ﻿choose between different femoral head sizes and materials, femoral neck lengths, stem sizes and taper characteristics to select the components that best fit the needs of the patient. Among other influencing factors, the variability in stem taper surface topography between hip stem designs has been identified as a potential cause of degradation by fretting and corrosion. However, so far, studies mostly compared smooth and threaded surface finishes, with no subdivision between different surface characteristics of threaded stem tapers^21–27^. While the taper connection strength is only slightly affected by the stem taper surface topography^45^, it remains understudied how the degree of corrosion and fretting is affected by varying stem taper thread profiles.

The strong variability in surface topography between different stem tapers has been pointed out in previous studies. Notably, Mueller et al. demonstrated that stem taper characteristics largely differ between manufacturers^36^. They assessed eight stem taper designs, including the Bicontact and SL-Plus that were also part of this study, and found larger variations in topographic parameter than in geometric parameter. Specifically, they reported the surface finish to be either smooth or threaded, with thread heights ranging from 2.63 to 49.48 µm. Here, the average profile heights reported for unused Bicontact and SL-Plus stem conform with our findings from retrieved implants. The largest thread heights were documented in Fitmore stems that are also manufactured by Zimmer, as are the Alloclassic and CLS stem characterized in the present study. However, the quantified thread heights in the Alloclassic and CLS stem are considerably larger compared to the Fitmore stem stressing that taper finishes are not comparable even when produced by the same manufacturer. Still, it should be noted that two different measurement methods were used: while a tactile surface roughness measurement instrument was utilized in the above-mentioned study, the surface topography was quantified optically in the present work. Therefore, it cannot be fully excluded that the choice of measurement technique may account for small variations in topographic parameters. Munir et al. characterized eleven stem tapers where four had a smooth surface while the remaining seven showed a surface finish with thread heights much lower than described in this study (13.5 µm, Corail)^21^. They reported a periodic difference in the repetition and heights of threads for three stem designs. This finding could not be verified in the current study where all stem tapers had a periodic thread profile. Previous studies reported the surface topography as sinusoidal despite the variations in thread height^21,22^. Here, we have shown that the surface between stem tapers also differs in the profile shape: the Alloclassic and CLS stems present a trapezoidal shape while the Bicontact and SL-Plus stem are characterized by a sawtooth and triangular shape. Brock et al. reported higher volumetric wear rates in shorter 12/14 tapers with a threaded surface finish (Corail) compared to longer and smooth 11/13 tapers (S-ROM) when mated with CoCr heads^39^. In conformity with these findings, Hothi et al. also found higher material loss rates in shorter and rougher Corail stems compared to longer and smoother S-ROM stems in metal pairings^38^. However, the influence of different surface topographies on wear rates in 12/14 stem tapers were not investigated in both studies, and the reported thread height of 14 µm in Corail tapers was much lower compared to the thread heights measured for the Alloclassic and CLS stem in the present work. The findings of Kop et al. conform with these observations as they reported that CoCr stems with a micro-grooved finish were more corroded compared to smooth finished stems^37^. Our study extends these findings as we found no significant differences in corrosion and fretting scores between lightly and deeply threaded tapers in metal pairings suggesting that, while a differentiation between smooth and grooved tapers might result in higher wear, the extent of surface threading does not affect the degree of degradation.

In addition to the surface topography, the material properties of the femoral head have been pointed out to affect implant performance. Ceramic femoral heads are valued owing to their positive wear properties at the bearing surface. Moreover, they are chemically inert and electrically insulating^46^, even though other studies question their chemical stability and reported surface degradation potentially induced by metal contamination^47,48^. Due to its insulating properties, when a head-neck interface is formed with a ceramic head, only the metal surface contributes to the formation of corrosion suggesting that corrosion should be minimized. In line with this, previous studies have stated that the use of ceramic heads can reduce, but not eliminate, corrosion at the head-neck interface^28,41,49^. Kurtz et al. applied the Goldberg scoring scheme to elucidate the influence of the femoral head material on fretting and corrosion damage^28^. Here, they reported that degradation also occurs with ceramic heads and stated that the mechanisms of fretting corrosion between ceramic and metal heads cohorts are analogous. Specifically, they compared fretting and corrosion scores in stems fabricated from TMZF, Ti-6Al-4V and CoCr mated with CoCr and ceramic heads and stated lower scores in ceramic heads compared to CoCr heads. Within the ceramic head group, they found the stem material, flexural rigidity and the body weight to be the most important factors predicting increased fretting and corrosion scores. Our results show no statistical difference in corrosion or fretting scores between metal and ceramic pairings with simultaneous consideration for the surface profile. However, differences may arise from the different adaptation of the scoring scheme: while Kurtz et al. assigned a single score to each taper that contained both fretting and corrosion phenomena, we assumed the adaptation of Triantafyllopoulus et al. and scored fretting and corrosion separately and in eight regions. Additionally, even though differences in surface topography were reported by Kurtz et al., they were not quantitatively assessed and only accounted for by matching stem manufacturers. However, it has also been reported that ceramic and metal heads perform equally with respect to corrosion and material loss, at least for CoCr alloy V40 stem tapers^50^. In our study, no significant differences in both fretting and corrosion were verified when comparing equal surface finishes. Although the statistical model indicated non-significant differences (*p* = 0.052) when comparing corrosion scores between metal and ceramic heads for lightly threaded stem tapers, this could be due to the low sample size. Arnholt et al. investigated a cohort of 120 stem tapers of different designs made of both Ti and CoCr alloys and concluded that the variations between smooth and micro-grooved stem tapers are not associated with fretting and corrosion damage^22^. Our results indicate a relationship between thread height and both corrosion and fretting scores when comparing lightly and deeply threaded profiles while differentiating between metal and ceramic head combinations. Lightly threaded tapers coupled with ceramic heads show less corrosion and fretting damage compared to higher thread heights. We speculate that the wide differences in taper surfaces can influence the clinical outcome in THAs. During assembly, the actual contact area between the stem taper and the head taper is limited to only a fraction of the nominal contact area due to the threaded surface topography. Witt et al. reported that low assembly forces result in regions of limited contact in micro-threaded stem tapers, and that even high assembly forces lead to actual contact areas of less than 20% of the overlapping interface area^33^. As a result, a crevice geometry between the asperities of the stem and neck taper is created that is then infiltrated by body fluids. While any modular connection, even ceramic pairings, will show some corrosion when exposed to body fluids and micromotion, the severity might be affected by the crevice volume^28,41,51,52^. The degree of crevice corrosion depends on the crevice volume and, hence, the volume of fluid present. The crevice formed by the head and stem taper can lead to limited fluid access resulting in changes in solution chemistry which can accelerate the formation of corrosion^53^. Smaller volumes at an occluded interface are more susceptible to crevice corrosion as oxygen diffusion into the crevice is restricted^54,55^. Contrarily, deeply threaded stem tapers result in larger crevice volumes and greater oxygen diffusion, making the interface less prone to corrosion. Consequently, corrosion would be expected to form earlier in interfaces with lightly threaded stem tapers where oxygen is depleted more quickly. However, it should be noted that the referenced studies regarding the formation of crevice corrosion investigated pairings with metal heads only. In the present study, a higher degree of corrosion could not be verified in lightly threaded compared to deeply threaded tapers coupled with metal heads. This could be indicative of the fact that corrosion might form earlier in smaller occluded volumes, but affects larger occluded volumes, once formation is initiated, more rapidly in the further course. This is in partial agreement with the results presented by Pourzal et al. who found that stem tapers with larger thread height coupled with metal heads had less severe corrosion and fretting damage in the short term but that damage gradually increased with prolonged implantation time, whereas smoother stem tapers resulted in a steep initial increase in damage scores followed by only a slight increase over time^26^**.** Interestingly, however, we found significantly less corrosion in lightly threaded compared to deeply threaded tapers mated with ceramic heads (and also compared to deeply threaded tapers coupled with metal heads) which indicates that different mechanisms might be at play for ceramic couplings. Even though no significant degradation would be expected in ceramic head pairings due to a greater corrosion resistance, such degradation has been documented in previous studies^28,41,56^. However, the exact underlying mechanisms of corrosion formation in ceramic compared to metal pairings still remain unknown. While it has been stated that the basic mechanism is similar for both material pairings (except that, with ceramic heads, only the male metal taper is involved in the oxide abrasion and repassivation process)^28^, it was also noted that a chemomechanical softening of ceramic heads with simultaneous hardening of the male taper due to oxidation might lead to increased degradation of the interface^57^. Also considering the reported surface instability of ceramic heads^48^, it becomes clear that further studies are needed to better understand the operating damage modes in ceramic head pairings that go beyond the scope of this study. Nonetheless, our results indicate that the stem taper surface topography plays a contributing role and should be considered in future studies.

In addition, relative micromotion between the stem taper and head taper under cyclic stresses during gait can affect the formation and degree of corrosion by disrupting the protective passive oxide film of the metal stem taper. However, the impact of surface threads on implant stability remains unclear due to complex contact mechanics at the head-neck junction. Falkenberg et al. reported equal amounts of micromotion for both smooth and micro-grooved stem tapers under varus and valgus loading^58^. However, computational studies considering microgrooves with thread heights below 14 µm came to dissimilar conclusions reporting that either smooth tapers provide better fixation^59^ and or that larger thread heights lead to higher contact pressures and implant stability^60^. In view of the high variability in surface topography presented in our study and considering that most studies do not consider the surface finish in detail (most often, they are referred to as either “smooth” or “rough”), we believe the stem taper surface topography to be an important parameter to report when assessing the taper performance and contact mechanics in future studies.

Furthermore, the flexural rigidity has been shown to affect the degree of fretting and corrosion as necks with a higher stiffness bend less resulting in less relative motion of the components^13^. Here, we found no association of flexural rigidity with fretting and corrosion scores. However, since the study was focused on 12/14 tapers fabricated from titanium alloys with similar Young’s moduli, the flexural rigidity in our cohort was in the limited range of 148.15 to 234.82 Nm^2^ whereas other studies investigated tapers with flexural rigidities ranging from 91.5 to 873.2 Nm^2^^13^. In agreement with other studies^16,28^ we found no correlation between time in situ and fretting and corrosion damage in both pairing types. Other influencing factors including different loading conditions, assembly techniques or the formation of a passivation layer with time may explain the missing correlation with the time in situ.

This study has some limitations. The femoral heads were not investigated in this study and no statement can be made about material loss that can occur in the head. However, it has been shown that both fretting and corrosion scores in the head and in the neck are correlated, making neck scores a good indicator of degradation in the head^13,16^. Additionally, corrosion and fretting scores were assessed in the head-neck contact region only, whereas previous studies evaluated the entire taper surface and also included non-contact regions in their assessment^13,40^. As less, if at all, fretting and corrosion damage is expected in non-contact regions, the exclusion of those areas would consequently result in relatively higher scores. Moreover, the surgeon-dependent impaction force is not known, which is known to affect the initial interlock strength^61^. This might further influence the degree of micromotion between the two surfaces facilitating increased fretting^62^ and corrosion damage by disruption of the passive film^56^. Also, as a retrospective study, the studied implants might not fully reflect functioning, non-revised implants. However, retrieval studies have been an established approach for in vivo characterization of implants in the past. Lastly, it is possible that corrosion damage may overlay fretting phenomena at the stem taper surface potentially causing an underestimation of the fretting damage. Albeit, this is an inherent limitation that cannot be avoided and has limited comparable studies as well.

In conclusion, this study shows that the stem taper surface topography varies considerably between different hip stem designs. The results from our study show that different threading profiles in metal and ceramic pairings affect corrosion and fretting scores within the modular head-neck interface. We found less corrosion damage in lightly threaded stem tapers coupled with ceramic heads compared to deeply threaded stem tapers coupled with both ceramic and metal heads. Also, fretting damage was significantly lower in lightly threaded compared to deeply threaded tapers in ceramic head pairings. Despite the multi-factorial nature of degradation at the head-neck interface, our data indicates that the thread height plays a relevant role, and that lightly threaded taper surfaces are preferable with regard to fretting and corrosion mitigation at least in combination with ceramic heads. In summary, the choice of material pairing and both the taper geometry and topography is very complex and dependent on numerous factors. Our results suggest that smaller taper threads result in less fretting and corrosion when using a ceramic femoral head but also underline the need that additional studies are needed to better comprehend the influence of different taper designs on degradation at the head-neck interface. Understanding the impact of different stem taper surface topographies on implant performance could help to improve the issue of corrosion and fretting damage in modern THA and lead to longer-lasting clinical results.
